# Multivariate Analysis of Dopaminergic Gene Variants as Risk Factors of Heroin Dependence

**DOI:** 10.1371/journal.pone.0066592

**Published:** 2013-06-28

**Authors:** Andrea Vereczkei, Zsolt Demetrovics, Anna Szekely, Peter Sarkozy, Peter Antal, Agnes Szilagyi, Maria Sasvari-Szekely, Csaba Barta

**Affiliations:** 1 Department of Medical Chemistry, Molecular Biology and Pathobiochemistry, Semmelweis University, Budapest, Hungary; 2 Institute of Psychology, Eötvös Loránd University, Budapest, Hungary; 3 Technical University of Budapest, Measurement and Information Systems, Budapest, Hungary; 4 3rd Department of Internal Medicine, Research Laboratory, Semmelweis University, Budapest, Hungary; Democritus University of Thrace, Greece

## Abstract

**Background:**

Heroin dependence is a debilitating psychiatric disorder with complex inheritance. Since the dopaminergic system has a key role in rewarding mechanism of the brain, which is directly or indirectly targeted by most drugs of abuse, we focus on the effects and interactions among dopaminergic gene variants.

**Objective:**

To study the potential association between allelic variants of dopamine D2 receptor (DRD2), ANKK1 (ankyrin repeat and kinase domain containing 1), dopamine D4 receptor (DRD4), catechol-O-methyl transferase (COMT) and dopamine transporter (SLC6A3) genes and heroin dependence in Hungarian patients.

**Methods:**

303 heroin dependent subjects and 555 healthy controls were genotyped for 7 single nucleotide polymorphisms (SNPs) rs4680 of the COMT gene; rs1079597 and rs1800498 of the DRD2 gene; rs1800497 of the ANKK1 gene; rs1800955, rs936462 and rs747302 of the DRD4 gene. Four variable number of tandem repeats (VNTRs) were also genotyped: 120 bp duplication and 48 bp VNTR in exon 3 of DRD4 and 40 bp VNTR and intron 8 VNTR of SLC6A3. We also perform a multivariate analysis of associations using Bayesian networks in Bayesian multilevel analysis (BN-BMLA).

**Findings and conclusions:**

In single marker analysis the *Taq*IA (rs1800497) and *Taq*IB (rs1079597) variants were associated with heroin dependence. Moreover, –521 C/T SNP (rs1800955) of the DRD4 gene showed nominal association with a possible protective effect of the C allele. After applying the Bonferroni correction *Taq*IB was still significant suggesting that the minor (A) allele of the *Taq*IB SNP is a risk component in the genetic background of heroin dependence. The findings of the additional multiple marker analysis are consistent with the results of the single marker analysis, but this method was able to reveal an indirect effect of a promoter polymorphism (rs936462) of the DRD4 gene and this effect is mediated through the –521 C/T (rs1800955) polymorphism in the promoter.

## Introduction

Substance abuse presents a major health and social problem worldwide. It is broadly accepted that multiple genetic and environmental risk factors and their interactions may contribute to the development of drug addiction. However, at present, little is known about the exact nature of these genetic components. Family-, twin- and adoption studies have shown that the heritability of alcoholism is around 40–60% [1], other studies found similar heritability values in the context of substance use [2], [3], [4]. Neurobiological models accentuate the key role of the brain’s reward system via the dopaminergic mesocorticolimbic pathway, which is modulated by complex, mutual interactions with other stimulatory and inhibitory neurotransmitter systems. Various drugs of abuse act at different points of these systems, but eventually they all converge into elevated levels of dopamine released in the nucleus accumbens [5], [6].

The dopaminergic system has a key role in the rewarding and reinforcing mechanisms of the brain [7], which is the most likely target of drug abuse. Dopamine can be released as a result of using drugs such as nicotine, cocaine, cannabis and opiates. After heroin is converted into morphine, it acts as a µ-opioid receptor agonist. Ligand binding decreases the release of GABA from interneurons, thus reducing the inhibitory effect of GABA on dopaminergic neurons and as a result a sustained synaptic level of dopamine can be observed, which is perceived as euphoria [8]. As interindividual differences in the function of the mesocorticolimbic dopaminergic reward system have an influence on the drug-induced response of the body, genetic variants of the neurotransmitter systems involved in the reward system - especially dopamine - are the most likely candidate genes of drug addiction.

Genetic polymorphisms of the dopaminergic system include single nucleotide polymorphisms (SNPs) and length polymorphisms of the receptors, transporters and metabolizing enzymes. One of the most widely studied candidate genes is the dopamine D2 receptor (DRD2) characterized previously with *Taq*IA, *Taq*IB, and *Taq*ID SNPs based on restriction digestion of this region with the *Taq*I enzyme. Early studies indicated the A1 allele of *Taq*IA as a risk factor of substance abuse, alcoholism [9] and heroin dependence [10], but some studies have failed to replicate these findings [11], [12]. Later it turned out that the *Taq*IA restriction fragment length polymorphism (RFLP) is located approximately 10 kilobases downstream from the DRD2 gene, in exon 8 of the ANKK1 (ankyrin repeat and kinase domain containing 1) gene [13], which is a member of the serine/threonine kinase family. The *Taq*IA polymorphism, causing an amino acid change in ANKK1 (Glu713Lys), seems to have a significant effect on the specificity of substrate binding. The protein product of the ANKK1 gene was considered as a negative regulator of the NF-κB (Nuclear Factor-KappaB) transcription factor [14]. Moreover, the expression level of NF-κB-regulated genes was shown to be altered by *Taq*IA variants in an in vitro luciferase system [15]. Since DRD2 is regulated by NF-κB [16], [17] it could be assumed that this ANKK1 variant can indirectly affect DRD2 receptor density. It is also possible, however, that the *Taq*IA SNP is only a marker of other functional DRD2 variants associated with addiction, such as the strongly linked *Taq*IB [18].

An interesting hypothesis has arisen from the possible role of decreased dopamine receptor density resulting in Reward Deficiency Syndrome [5]. It is well known that under normal conditions dopamine is released into the synapse, binds to dopamine receptors, inducing euphoria and stress reduction. Reward Deficiency Syndrome is characterized by a lower basal dopamine level due to insufficient receptor capacity resulting in a need of a certain amount of dopamine to feel good (this could be achieved by rewarding experiences such as drugs, gambling, alcohol, etc.). Studies on animal models further underline the role of DRD2 in drug addiction as the rewarding effects of opiates were found absent in mice lacking the D2 receptor gene [19], [20].

Another widely studied polymorphic dopamine receptor is the dopamine D4 receptor gene (DRD4) with more than 200 SNPs and several VNTRs. The 48 bp repeat polymorphism is an exonic variant (48 bp VNTR) changing the length of the third intracellular loop of the receptor with a possible effect on signaling efficiency [21]. The DRD4 7 repeat allele seems to show decreased sensitivity to dopamine compared with the 4 repeat allele [22] and according to recent neurobiological findings it does not form heteromers with D2 receptors in the striatum [23]. Carriers of the “long” allele possessing 7 repeats of the 48 bp sequence were shown to have higher novelty seeking scores compared to non-carriers assessed by psychological questionnaires [24], [25], [26], [27], however, replication studies were contradictory (for a review see Kluger et al., 2002 [28]). It has also been shown that individuals with high novelty seeking scores are prone to increased substance use [29], [30], [31]. Therefore, the DRD4 long allele was considered as a predictive marker of various addictive behaviors. This hypothesis was supported in alcoholics [32], heroin dependent patients [33] and individuals with eating disorders [34], but subsequent replications were also contradictory (for a review see McGeary, 2009 [35]). One of the possible reasons for unsuccessful replication studies might be the high genetic variability of the DRD4 gene including other functional variants beside the exonic 48 bp VNTR. Molecular effect of DRD4 promoter region polymorphisms (120 bp duplication, −615 A/G, −616 C/G and −521 C/T) has been studied by others, as well as in our laboratory [36], [37], [38], [39], [40], [41]. Furthermore we previously described a novel SNP −615 A/G generating a source of possible misgenotyping of the adjacent SNP −616 C/G by conventional methods [42].

Catechol-O-methyl transferase (COMT) plays an important role in the regulation of synaptic dopamine levels since it is responsible for the catabolism of dopamine. The Val^158^Met exonic SNP (rs4680) of COMT is considered to influence enzyme activity [43], making this polymorphism a possible marker of genetic predisposition to addiction [44].

The dopamine transporter is responsible for the re-uptake of dopamine from the synapse in midbrain dopaminergic neurons [45], playing a key role in the homeostatic regulation of dopaminergic neurotransmission. Genetic variants of the dopamine transporter gene (SLC6A3 or commonly DAT) have also been implicated in human mental disorders such as parkinsonism, Tourette syndrome, and substance abuse [46].

Here, we present a combined analysis of the key candidate polymorphisms in the dopaminergic system as possible risk factors for heroin dependence in a case-control study of Central European descent (Hungarian). Moreover, we focus on the interactions among these genetic variants using Bayesian networks in Bayesian multilevel analysis (for previous applications of BN-BMLA, see [47], [48], [49], [50]).

## Materials and Methods

### 1. Subjects

#### Cases

The initial cohort consisted of 307 heroin dependent patients. Four subjects suffering from a major psychiatric disorder (schizophrenia and major depression) with possible involvement of the studied neurotransmitter systems were excluded from the study. All subjects were unrelated with Hungarian origin. The final sample included 303 heroin dependent patients (211 (69.6%) males and 92 females (30.4%)) from three centers, the National Institute of Psychiatry and Neurology, Budapest; the Dr. Farkasinszky Terézia Youth Drug Centre, Szeged and the Nyírő Gyula Hospital Drug Outpatient and Prevention Center, Budapest.

Diagnosis was made based on DSM-IV criteria (American Psychiatric Association, 1994). The age of subjects at diagnosis was 15–70 years (mean age = 28.64 years ±6.448). All patients with heroin dependence reported heroin as their primary drug of choice. Non-opioid substance use (such as alcohol, cocaine, marijuana, amphetamine, LSD, benzodiazepine, etc.) was not an exclusion criterion since heroin dependent patients frequently use alcohol, cannabis or cocaine as secondary drugs.

#### Controls

Genotype data of 555 sexually matched healthy Hungarian control subjects (386 [69.5%] males and 169 females [30.5%]) was used to construct a large normative sample for determination of control genotype and allele frequencies. These subjects did not suffer from psychiatric disorders. Nicotine dependence was not an exclusion criterion from the control group.

The research protocol has been approved by the Hungarian National Ethical Committee (TUKEB), as well as the Ethical Board of Semmelweis University and the Institute of Psychology, Eötvös Loránd University. All participants were over the age of 18 at the time of inclusion in the study. All participants provided written informed consent.

### 2. Genotyping

A non-invasive DNA sampling method was used to obtain the sufficient quantity of buccal cells (method is described elsewhere [51]). Genomic DNA was isolated from buccal swabs [51] or mouthwash using the DNA-purification kit obtained from Gentra (Minneapolis, US) and approximately 1 ng DNA was used as template for each of the tested polymorphisms, performed as described earlier (*DRD4 gene:* −521 C/T SNP [52], 120 bp duplication [40], DRD4 VNTR [53]). The −521 C/T polymorphism was determined by two independent methods using a newly designed primer pair [54], and only genotypes with identical results were accepted. Using this improved method the genotype distribution of the control population corresponds to the Hardy-Weinberg equilibrium (see Table 1).

**Table 1:** Results of the case-control analysis.

Gene	Marker	Genotype	Control(N)	Control(%)	HWE	Case(N)	Case(%)	HWEp-value	Dominant modelp-value	Dominant model OR(95% CI)
**COMT**	Val158Met	GG	125	22.5%	0.99	75	24.8%	0.993	0.329	1.17 (0.85–1.62)
	rs4680	AG	275	49.6%		152	50.4%			
		AA	155	27.9%		75	24.8%			
**ANKK1**	*Taq*IA	TT	17	3.2%	0.912	18	6.1%	0.757	**0.009**	1.49 (1.11–2.00)
	rs1800497	CT	148	27.9%		100	34.0%			
		CC	366	68.9%		176	59.9%			
**DRD2**	*Taq*IB	AA	12	2.3%	0.833	13	4.6%	0.869	**0.003** *	1.61 (1.18–2.21)
	rs1079597	AG	123	23.5%		88	31.3%			
		GG	388	74.2%		180	64.1%			
	*Taq*ID	CC	78	15.8%	0.989	56	19.4%	0.898	0.381	1.15 (0.84–1.56)
	rs1800498	CT	239	48.2%		138	47.8%			
		TT	178	36.0%		95	32.8%			
**DRD4**	−521 C/T	CC	111	21.0%	0.989	61	20.2%	0.282	**0.007**	0.66 (0.48–0.89)
	rs1800955	CT	278	52.6%		134	44.4%			
		TT	140	26.4%		107	35.4%			
	−615 A/G	GG	4	0.8%	0.18	5	1.7%	0.731	0.302	1.18 (0.86–1.63)
	rs936462	AG	128	24.2%		80	26.6%			
		AA	395	75.0%		215	71.7%			
	−616 C/G	GG	120	22.5%	0.927	62	20.5%	0.307	0.610	1.09 (0.79–1.50)
	rs747302	CG	271	50.7%		164	54.3%			
		CC	143	26.8%		76	25.2%			
	120dup	1-absent	392	70.9%	0.974	211	69.6%	0.851	0.702	1.06 (0.78–1.44)
		1-present	161	29.1%		92	30.4%			
	48 bp VNTR	7-absent	337	62.3%	0.219	195	64.8%	0.977	0.472	0.90 (0.67–1.20)
		7-present	204	37.7%		106	35.2%			
**SLC6A3**	40 bp VNTR	9-absent	275	51.2%	0.998	164	56.2%	0.928	0.092	0.78 (0.59–1.04)
		9-present	262	48.8%		128	43.8%			
	intron 8 VNTR	2-absent	343	63.3%	0.852	195	64.8%	0.108	0.664	0.94 (0.70–1.26)
		2-present	199	36.7%		106	35.2%			

HWE: p value of deviation from Hardy-Weinberg equilibrium.

OR: Odds ratio, CI: confidence interval.

* significant after Bonferroni correction: p<0.0045 (0.05/11) for 11 tests.

Genotyping methods included restriction fragment length polymorphism, allele-specific amplification and real-time PCR. Genotyping procedures for the DRD2/ANKK1 *Taq*IA (rs1800497), the DRD2 *Taq*IB (rs1079597) and *Taq*ID (rs1800498), the SLC6A3 40 bp VNTR in the 3′ untranslated region, the DRD4 48 bp VNTR in exon 3 and 120 bp duplication in the promoter region, and the allele-specific amplifications of the DRD4 −616 C/G (rs747302), −615 A/G (rs936462), −521 C/T (rs1800955) and COMT Val158Met (rs4680) were carried out using published protocols [53], [55], [56], [57], [58], [59], [60]. The genotype-phenotype data underlying the present study was deposited in the Dryad Data Repository (www.datadryad.org) at http://dx.doi.org/10.5061/dryad.975 kk.

#### 2.1. Restriction fragment length polymorphism

1 µM of each primer was used to carry out the amplification (method developed by Percy et. al. [52]). In the case of COMT the forward (5′-CTC ATC ACC ATC GAG ATC AA-3′) and reverse primers (5′-CCT TTT TCC AGG TCT GAC AA-3′) were used with 1x Buffer and Q-solution (Qiagen); 200 µM dATP, dCTP, dGTP and dTTP; 0.1 U HotStarTaq DNA polymerase and 5 ng genomic DNA (10 µL final volume). Thermocycling conditions: 95°C for 15 minutes, 40 cycles of 94°C, 30 sec denaturation at 52°C, 30 sec annealing and 1 min at 72°C for extension and finally a 10-minute polymerization at 72°C. Digestion was carried out using *Nla*III restriction enzyme. In case of DRD2 we had three SNP-RFLP polymorphisms: the *Taq*IA (DRD2/ANKK1), *Taq*IB and *Taq*ID. For PCR we used the same method with specific primer pairs. *Taq*IA: forward: 5′-CCG TCG ACG GCT GGC CAA GTT GTC TA-3′, reverse: 5′-CCG TCG ACC CTT CCT GAG TGT CAT CA-3′. *Taq*IB: forward: 5′-GAT ACC CAC TTC AGG AAG TC-3′, reverse: 5′-GAT GTG TAG GAA TTA GCC AGG-3′. *Taq*ID: forward: 5′-CCC AGC AGG GAG AGG GAG TA-3′, reverse: 5′-GAC AAG TAC TTG GTA AGC ATG-3′. For the genotype dependent digestion 1x *Taq*I Buffer, 2.5 U Taq restriction enzyme, 5 µL PCR product supplemented with distilled water to reach the final volume of 10 µL. Digestions were performed during a 65°C overnight incubation followed by a fragment separation on 2.5% agarose gel matrix.

#### 2.2. Allele-Specific Amplification (ASA)

In the case of COMT we used an ASA by tetra-primer PCR. A pair of flanking primers (1: 5′-TGC TCA CCT CTC CTC CGT-3′, 2: 5′-ACA CCC ATA CAA GCA TTC ATC-3′) and two internal primers (1: 5′-CAC ACC TTG TCC TTC AC-3′, 2: 5′ TGG TGG ATT TCG CTG GCA 3′).

DRD4 –521 C/T genotypes were also determined by ASA. Flanking primers are the following: 5′-GGA ATG GAG GAG GGA GCG GG-3′; 5′-CGC TCC ACC GTG AGC CCA GTA T-3′. Internal specific primers: 5′-GGA GCG GGC GTG GAG GGC-3′; 5′-GCC TCG ACC TCG TGC GCA-3′. Same thing with the −616 C/G SNP. Flanking primers: 5′-GAA CCT ACC CCG GCC TGT CGT-3′; 5′-AGA CGG GAA TGA AGC GAG GTG G-3′. Internal primers: 5′-TGG TCG CGG GGG CTG AGC-3′; 5′-CCC CCC MGC AGC CTC TGG YC-3′ (M = A+C, Y = C+T, degenerated nucleotides).

As for genotyping of the intron 8 VNTR of the DAT gene we used a PCR-based method with forward (5′-GCTTGGGGAAGGAAGGG-3′) and reverse primers (5′-TGTGTGCGTGCATGTGG-3′). The same method was used for genotyping the DAT1 40 bp VNTR in the 3′ untranslated region of the gene: forward primer: 5′-TGT GGT GTA GGG AAC GGC CTG AG-3′, reverse primer: 5′-CTT CCT GGA GGT CAC GGC TCA AGG-3′. In the case of the VNTR polymorphisms of the DRD4 gene: the 120 bp duplication primers: forward primer: 5′-GTT GGC TGT CTT TTC TCA TTG TTT CCA TTG-3′, reverse primer: 5′-GAA GGA GCA GGC ACC GTG AGC-3′; the 48 bp VNTR primers: forward primer: 5′-GCG ACT ACG TGG TCT ACT CG-3′, reverse primer: 5′-AGG ACC CTC ATG GCC TTG-3′.

### 3. Statistical Analyses

Our case-control analysis applied a dominant model of minor alleles, i.e. genotypes were grouped according to the presence or the absence of the minor allele. For single marker analysis genotype frequencies were compared by Chi-squared test, using SPSS 17.0 for Windows (SPSS Inc., Chicago, IL, USA). P-values<0.05 were considered nominally significant. Correction for multiple testing was made according to the stringent Bonferroni correction: the threshold significance was adjusted to p<0.0045, where 0.0045 = 0.05/11 (since 11 polymorphisms were tested).

Linkage disequilibrium between the ANKK1 and DRD2 SNPs (*Taq*IA and *Taq*IB) was calculated by the HaploView program, version 4.2. (Daly Lab, Cambridge, MA, USA) [61]. Haplotype analyses were conducted by likelihood-based association analysis using UNPHASED [62].

Besides standard association testing we performed a systems-based association analysis, as well. A central concept in this approach is strong relevance. A variable is strongly relevant if its statistical association remains relevant given all the other variables, i.e. its effect is not mediated by other variables [63]. This concept can be generalized to multiple variables as follows. A set of variables is sufficient as explanatory variables for a given target if all the other variables are not statistically associated, i.e. if these variables shield the target from the effect of the other variables [64]. This set of variables ***X*** *’* (a subset of all variables ***X***) is called a Markov blanket set (MBS(*Y*)) of variables with regard to target ***Y***. For a given variable/factor *X_i_*, being a member of MBS is called Markov blanket membership (MBM(*Y*, *X_i_*)). The Bayesian framework allows us to calculate the posterior probabilities of these properties by performing a random walk over the set of possible network structures using a Markov chain Monte Carlo simulation. We used the method published earlier to calculate the MBM and MBS posterior probabilities, with the following settings: 8*10^6^ MCMC steps, 10^6^ burn-in steps, 5 maximum parents, Cooper-Herskovits prior (for the detailed description of the approach, see [47]).

These posteriors offer a new way to quantify the interaction or redundancy of the variable set {*X_i1_*, …, *X_in_*} over dataset *D*. Redundancy shows us if the effect of a set of variables can be accounted for by any of the variables in the set, while interaction shows us effects, which are greater than the effect of the individual variables. We computed the interaction and redundancy scores *R* for pairs of predictors *X_i_, X_j_* with the following formula:
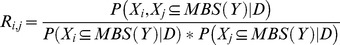



By applying this method, we can see whether the differences of *R* values from 1 indicate an interaction (*R*>1) or a redundancy (*R*<1). The corresponding Interaction Ratio is then defined by *IR* = ln(*R*), and the Redundancy Ratio by *RR* = ­ln(*R*).

## Results

### 1. Single Marker Analysis

A sample of 303 heroin dependent patients and 555 controls of Central European descent were analyzed for 7 SNPs and 4 VNTRs of the dopaminergic system. Main characteristics of SNPs and VNTRs studied in this work are summarized in Table 2 and 3, respectively. Table 2 shows the gene symbols, alternative symbols, genomic coordinates, rs numbers and alternative names of SNPs studied here. Minor allele frequencies (MAF) of our own data are compared with data of mixed European population from the ALFRED (The ALlele FREquency Database - http://alfred.med.yale.edu/) database. Table 3 contains the basic properties of the length polymorphisms possessing variable numbers of tandem repeats (VNTRs) assessed in this study.

**Table 2:** Genetic variants of the dopaminerg system.

Single nucleotide polymorphisms								
Gene symbol and name**		Chromosomal location	Genomic coordinates	rs number	Alternative names	In gene location	MAF*	MAF in the present study
**COMT**	Catechol-O-methyltransferase	22q11.21	22∶19,929,262–19,957,497	rs4680	Val158Met	exon 3	0.558	0.470
**DRD2**	Dopamine receptor D2	11q23	11∶113,280,316–113,346,000	rs1079597	*Taq*IB	intron 1	0.163	0.140
				rs1800498	*Taq*ID	intron 2	0.360	0.400
**ANKK1**	Ankyrin repeat and kinase domain containing 1	11q23.2	11∶113,258,512–113,271,139	rs1800497	*Taq*IA	exon 8	0.190	0.170
**DRD4**	Dopamine receptor D4	11p15.5	11: 637,304–640,705	rs1800955	−521 C/T	5′ promoter	0.400	0.470
				rs936462	−615 A/G	5′ promoter	0.132‡	0.130
				rs747302	−616 C/G	5′ promoter	0.485‡	0.480

* MAF source: ALFRED database - Mixed European population.

** Approved symbol from the HUGO Gene Nomenclature Committee (HGNC) database.

‡ only Hungarian data was available.

**Table 3:** Genetic variants of the dopaminerg system.

Length polymorphisms						
Gene symbol and name**		Alternativenames	Chromosomallocation	Genomic coordinates	Repeat region	In gene location
**DRD4**	Dopamine receptor D4		11p15.5	11∶637,304–640,705	120 bp dup	1.2 kb upstream of the initiation codon
					48 bp VNTR	exon 3
**SLC6A3**	Solute carrierfamily 6(neurotransmitter transporter,dopamine),member 3	Dopamine transporter 1(DAT1)	5p15.33	5∶1,392,904–1,445,542	40 bp VNTR	3′ UTR
					intron 8 VNTR	intron 8

** Approved symbol from the HUGO Gene Nomenclature Committee (HGNC) database.

VNTR = Variable number of tandem repeats.

**Figure 1:**
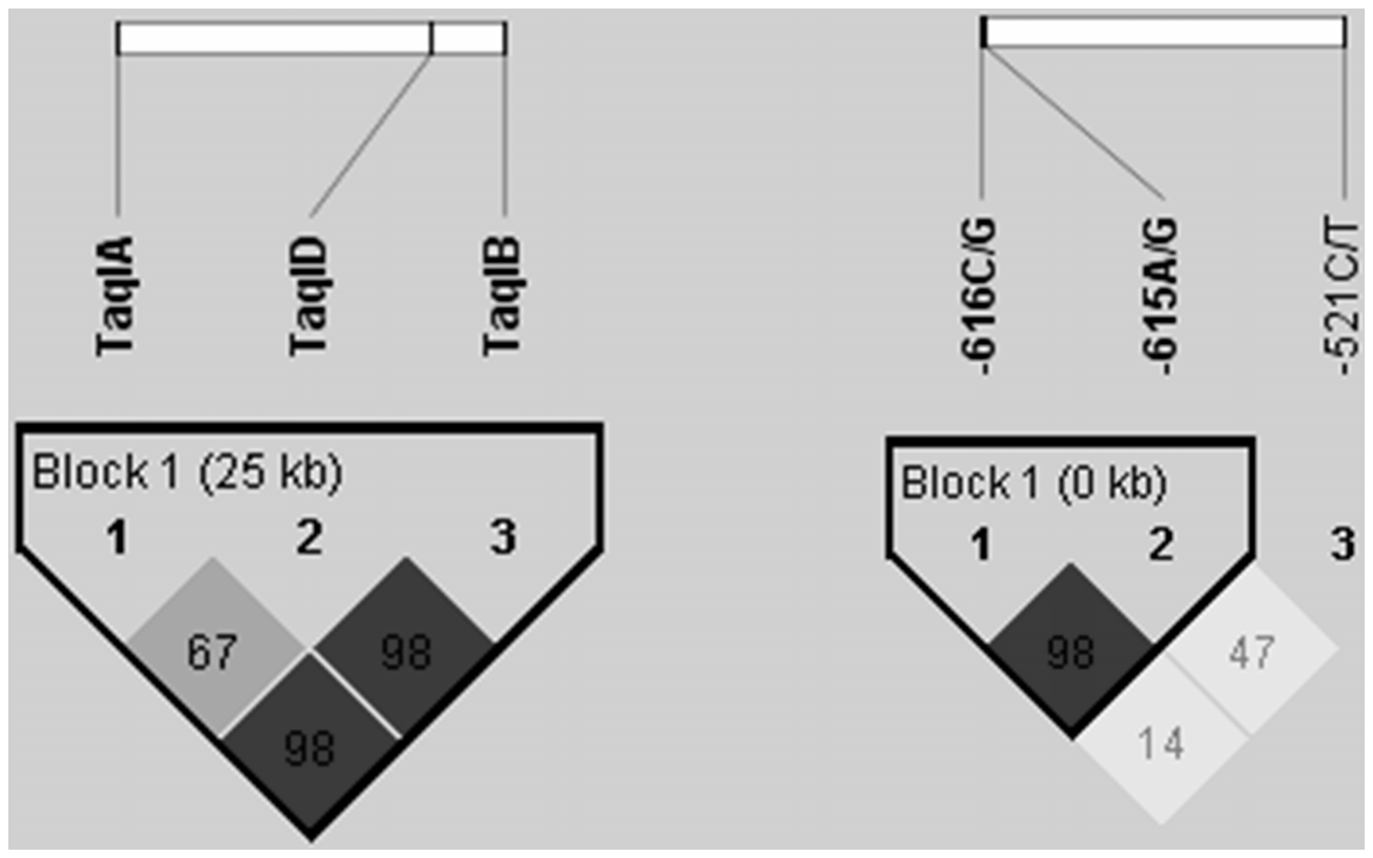
HaploView analysis of pairwise linkage disequilibrium in DRD2 and DRD4. Three marker SNPs are shown in both panel A for DRD2 and panel B for DRD4. The colors represent the relative D’/LOD scores. Linkage disequilibria are displayed as pairwise D’ values multiplied by 100. Shading represents the magnitude and significance of pairwise LD on a grey-scale (black–high LD; white–low LD).

For testing the linkage disequilibrium (LD) among multiple polymorphisms of DRD2 (Figure 1A) and DRD4 (Figure 1B) genes the HaploView program was used. A strong LD was found between the *Taq*IA and *Taq*IB (98%) in the DRD2 gene. Interestingly, the linkage of the third studied DRD2 SNP (*Taq*ID) lying in between the strongly linked SNPs (*Taq*IA and *Taq*IB) shows an ambiguous picture: it is linked strongly to *Taq*IB (98%) but not to *Taq*IA (67%). The two adjacent SNPs in the DRD4 gene located in the promoter region with 615 and 616 bp upstream of the start codon, are strongly linked (98%), as expected. On the other hand, the −521 C/T SNP has only a weak linkage to the previous ones (47%).

Results of our single marker case-control analysis are listed in Table 1. Both the common names and rs numbers of SNPs are given for better understanding. The table shows the number of the individuals (N) and also the percentages (%) in each genotype category for the control and for the heroin dependent (case) sample. Observed genotype frequencies are consistent with Hardy-Weinberg equilibrium (see the p values in column “HWE”) in the control group for all SNPs. In case of length polymorphisms genotype categories were grouped according to the presence or absence of the second most frequent allele and the HWE was calculated for these categories. The complete list of the VNTR genotypes including all the variants is shown in Table S1.

The frequentist calculation method of case-control analysis was based on the presence or the absence of the minor allele (dominant model), all p values showing nominal significance (p<0.05) are seen in bold in Table 1. According to the data the minor (T) allele containing genotypes (TT+CT) of *Taq*IA polymorphism (rs1800497) in the ANKK1 gene were overrepresented in patients (6.1%+34%) when compared to the controls (3.2%+27.9%) suggesting a genetic effect of the T allele on heroin dependence risk (p = 0.009). A similar effect was found for the strongly linked *Taq*IB SNP (rs1079597) of the DRD2 gene: the minor A allele containing genotypes were overexpressed among heroin dependent patients (4.6%+31.3%) compared to the control group (2.3%+23.5%, p = 0.003). As seen in Table 1, the odds ratios of these polymorphisms are relatively high (1.49 and 1.61 for *Taq*IA and *Taq*IB, respectively). Moreover, the –521 C/T polymorphism of the DRD4 gene and heroin dependence also showed a nominal association (p = 0.007). Although the two alleles have quite a similar frequency, the minor C allele seems to have a protective effect against heroin addiction as both CC and CT have a lower frequency among heroin dependent patients (20.2%+44.4%) than controls (21.0%+52.6%). No significant differences were found between heroin dependent patients and controls in the other polymorphisms included in this study. After applying the stringent Bonferroni correction for multiple testing (p<0.0045 [0.05/11] for the 11 tests), only the effect of the DRD2 *Taq*IB SNP remained significant (labeled by an asterisk in Table 1) suggesting that the presence of the DRD2 *Taq*IB minor allele is a strongly relevant component in the genetic background of heroin dependence.

### 2. Multiple Marker Analysis

Since the Bonferroni correction is one of the stringent methods for analyzing multiple comparison data, methods of multiple marker analysis were also applied in order to avoid false negative findings. As a first step, haplotype analysis was carried out for *Taq*I polymorphisms in the DRD2/ANKK1 genes and the results are presented in Table S2. Although we found nominally significant associations of haplotypes *Taq*IA-*Taq*IB, *Taq*ID-*Taq*IB and *Taq*IA-*Taq*ID-*Taq*IB (p = 0.0063, p = 0.0136, p = 0.028, respectively) with heroin addiction, these results might be a simple consequence of the strongly linked minor risk alleles.

The T∼A haplotype of the two strongly linked minor alleles of *Taq*IA and *Taq*IB SNPs was overrepresented in the case group (19.9%) compared to controls (13.8%), suggesting the association of T∼A haplotype with heroin dependence (p = 0.0063). It should be noted, however, that the total of the haplotype frequencies of linked minor alleles (T∼A: 13.8% in controls) and linked major alleles (C∼G: 82.9% in controls) is near 97%, and the other haplotypes are extremely rare. Therefore, the association of T∼A haplotype with heroin dependence might be a direct consequence of the associating minor alleles. Haplotype frequencies of DRD4 promoter SNPs were also calculated and compared between the control and heroin dependent samples but no significant differences were obtained (see: Table S3).

**Figure 2:**
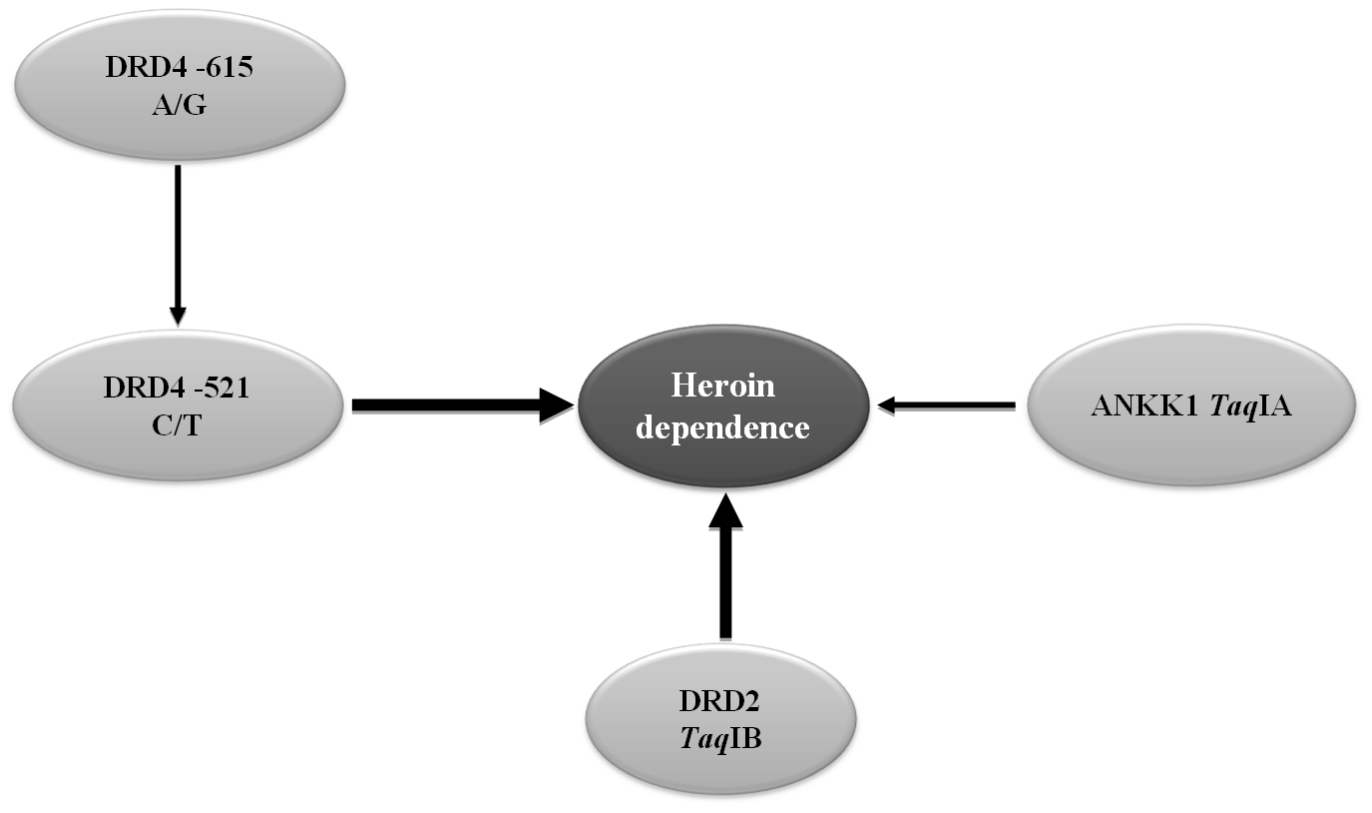
Posterior probabilities for variables in association with heroin use. In this graphic visualization each variable is represented by a grey oval, while the target variable is the black oval. Variables are nodes in Bayesian networks. The arrows between nodes represent the direct associations between variables; the thickness of the arrows reflects the posterior probability of the edge being present in the model. The DRD4 −615 A/G has no direct influence on the target variable, the effect is mediated through the DRD4 −521 C/T.

In an independent method for multiple marker analysis, we applied a Bayesian network based Bayesian multilevel analysis (BN-BMLA) in order to evaluate dopaminergic risk factors of heroin addiction. Using the same set of phenotype and genotype data as in the above calculations, we summarized the results in a consensus Bayesian network as seen in Figure 2. Our results suggest significant effects of ANKK1 (*Taq*IA), DRD2 (*Taq*IB) and DRD4 (–521 C/T) SNPs, which are consistent with the findings of the single marker analysis without correction for multiple testing, as seen earlier.

An additional interesting finding of the network analysis is the effect of another DRD4 promoter polymorphism (−615 A/G), which has an indirect effect on our target variable (heroin dependence). According to our results, the effect of the −615 A/G polymorphism is manifested through another DRD4 polymorphism, the −521 C/T. The combined effect of these SNPs was also seen by classical statistical methods, as shown in Table 4. No difference of the DRD4 −521 C/T genotype distribution was observed in the presence of the −615 G allele, while in the absence of this allele a significant (p = 0.0013) effect of the −521 C/T polymorphism was demonstrated. These data are in good agreement with the indirect effect of the DRD4 −615 SNP shown on Figure 2.

**Table 4:** The combined effect of the DRD4 −615 A/G and −521 C/T SNPs.

			DRD4 −521 C/T				p-value*	OR** (95% CI)
			C absent (TT)		C present (CC,CT)			
**DRD4 -615 A/G**	G-absent (AA)	control	114	21.9%	275	52.8%	**0.0013**	1.77 (1.25–2.50)
		addict	91	30.3%	124	41.3%		
	G-present (AG,GG)	control	23	4.4%	109	20.9%	1.0000	0.93 (0.45–1.93)
		addict	14	4.7%	71	23.7%		

* Pearson Chi-Square Exact Sig. (2-sided).

** OR: Odds ratio, CI: confidence interval.

**Figure 3:**
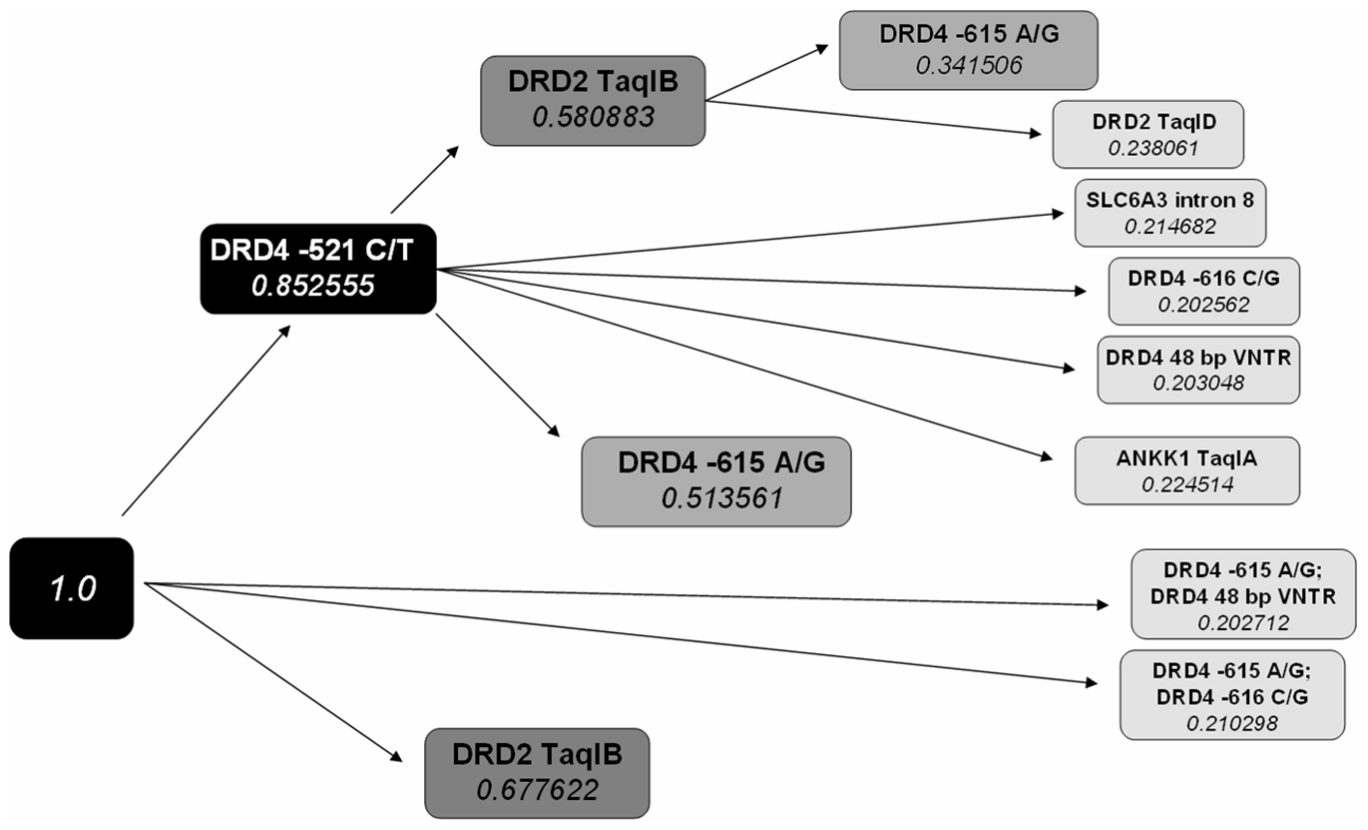
Dendrogram of sub-relevant sets of SNPs. This relevance tree shows the hierarchy of relevant variable subsets. Starting from left to right, paths starting from the (1.0) node show us the relevance of the subset of variables along the path. The respective posterior probability is shown in the lower part of the end node. The posterior probability of DRD4 −521 and DRD −615 both being relevant variables is 0.51. The posterior probability decreases when increasing the number of variables (nodes) in the model. The *Taq*IA polymorphism only enters the model when *Taq*IB is not present, signifying its redundancy.

Another interesting finding in the Bayesian analysis is the more pronounced effect of the *Taq*IB polymorphism compared to *Taq*IA. According to the results of the network analysis there is a strong redundant effect between the DRD2 and ANKK1 gene polymorphisms (Redundancy Ratio of 1.201). This means that the posterior probability of *Taq*IB or *Taq*IA being present alone in our model is more than 3.32 times as likely as both of them being present in the model. Our model yielded a higher posterior probability of relevance for the DRD2 *Taq*IB (P = 0.67), than the relevance of the ANKK1 *Taq*IA (P = 0.27) variants (Figure 3), therefore the effect of the *Taq*IA seems to be negligible compared to *Taq*IB. The probable multivariate models can be seen in Figure 3 by starting from the left (1.0) empty model. Each step from the root node of the dendrogram along the edges adds another variable to the model. The P value under the name of each node corresponds to the posterior probability of the model. The Bayesian network (Figure 2) can be achieved in 3 steps on the dendrogram. The detailed results of the Bayesian analysis are shown in Figure S1 showing our findings including relevance, redundancy and interaction of the studied polymorphisms.

## Discussion

Genetic variants of the dopaminergic neurotransmitter system as possible risk factors for heroin dependence have been extensively studied but results are contradictory. The subtelomeric region of chromosome 11p–where the DRD4 gene is located - was identified in a genome-wide search for quantitative trait loci influencing substance dependence vulnerability [65]. The DRD4 gene is rich in polymorphisms, including the one of the most extensively studied 48 bp repeat polymorphism in exon 3 (48 bp VNTR). Association of substance dependence and the 48 bp VNTR was first proposed by Kotler et al. [66] and replicated by others [67], [68], [69], but contradictory results were also obtained [70], [71], [72]. Our data presented here did not show any tendency of association between the 48 bp repeat and the risk of heroin dependence in a cohort of 303 cases and 555 controls of Central European descent. The promoter region of DRD4 is also highly variable, including the −521 C/T, the −616 C/G SNP and the −615 A/G variants described previously in our laboratory [42]. A previous association study between the −521 C/T polymorphism and heroin abuse conducted on 387 Chinese subjects [72] reported no significant difference of the −521 C/T polymorphism, although their data showed a small, but non-significant increase in frequencies of the −521 C allele and CC genotype in the injector subgroup. On the contrary, Lai and coworkers demonstrated a higher T allele frequency among heroin dependent patients (p = 0.0002) suggesting a higher preference for heroin use in individuals with DRD4 −521 TT genotype [73]. This is in good agreement with our present finding, which showed a nominal significance (p = 0.007) of the −521 TT genotype as a risk factor of heroin abuse. It also supports our results with heroin [74] and nicotine dependence [75] published earlier, as well as a recent study by others [76]. This effect of the −521 C/T polymorphism was no longer significant according to the frequentist model if corrected for multiple testing. Haplotype analysis of the promoter region did not yield any significant result either. On the other hand, using the multivariate model implying a Bayesian multilevel analysis we were able to confirm the association of −521 C/T polymorphism and heroin dependence, also demonstrating the indirect effect of −615 A/G. The presented results of the multilevel analysis of the DRD4 promoter polymorphisms clearly show the possible sources of contradictions obtained by the single marker approach, where interactions cannot be taken into consideration. Association of −521 C/T and Novelty Seeking, a risk factor of drug addiction, was also demonstrated by others and by our laboratory [77], [78]. The replication studies of this association were also contradictory as in most cases of single marker analysis, but a meta-analysis provided further support for the role of −521 C/T in Novelty Seeking [79]. These previous results seem to underline our positive findings on the −521 C/T SNP obtained by the Bayesian multilevel analysis rather than the negative results when applying the single marker method with correction for multiple testing.

The main results of our study are organized around the dopamine D2 receptor and the neighboring ANKK1 gene, encompassing the widely studied *Taq*IA polymorphism previously thought to belong to the DRD2 gene. In a Spanish study [80] the possible effect of the *Taq*IA polymorphism was examined in methadone-treated substance dependent patients, who marked heroin as their primary drug. Some of the subjects used other, non-opioid substances besides heroin, however these were not excluded from the study since DRD2 is potentially a non-specific genetic predictor of substance use and dependence. This case-control study reported a relationship between DRD2 and the potential risk of developing heroin dependence. Based on the results the A1A1 (rare) genotype of the *Taq*IA polymorphism is associated with heroin dependence regardless of gender, however the A1 allele showed association only in males. A Chinese study (2009) carried out on Han Chinese subjects found that the ANKK1 *Taq*I A1 allele carriers (genotypes A1A1 and A1A2) were prone to heroin abuse in models of dominance or co-dominance [10] in agreement with our findings. Results of association studies between the *Taq*IA polymorphism and substance use disorder has been summarized in a meta-analysis of Young et al (2004) evaluating the results of 55 previous studies [81]. They concluded that the A1 allele is a predictive marker of substance misuse and a partial marker of severe substance use disorders. The statistical power of this study was fairly strong at that time due to the sample size of ten thousand, making it a robust meta-analysis. Here we present a nominally significant association between the ANKK1 *Taq*IA SNP and heroin dependence (p = 0.009) which was no longer valid after the stringent Bonferroni correction of multiple testing. The negative finding between *Taq*IA and heroin dependence was confirmed by our multilevel analysis, as well, supporting the importance for multiple correction in this case.

Unlike the widely studied *Taq*IA, there are only a few association studies with the *Taq*IB SNP, located in intron 1 of the DRD2 gene and substance-related disorders. The possible role of *Taq*IB was shown in nicotine addiction [82], cocaine-dependence [83] and psychostimulant abuse [84], but significant association with heroin dependence has not been published so far to the best of our knowledge. According to our single marker analysis presented here the *Taq*IB SNP is the only dopaminergic polymorphism, which remained significant (p = 0.003) after Bonferroni correction. Our multilevel analysis gave similar results, demonstrating that the *Taq*IB SNP of DRD2 gene has the highest level of relevance to heroin abuse, while a high redundancy or low relevance was found for the *Taq*IA SNP (Figure 3), a polymorphism in the neighboring gene, which is strongly linked to the studied DRD2 SNP. Based on these results we hypothesized that it is rather a DRD2 than an ANKK1 gene variant which has a direct effect on heroin dependence. One of the limitations of our study is that we assessed only two SNPs (*Taq*IB and *Taq*ID) of the DRD2 gene. A higher resolution linkage study might be necessary to reveal the functional variants of this very important receptor gene, including the study of the exonic Ser311Cys variant [85] shown as a risk factor for schizophrenia.

Genes playing a role in the transport (SLC6A3, or DAT1) and in the catabolism of dopamine (COMT) also seemed to be challenging candidate genes for heroin abuse. Two length polymorphisms of SLC6A3 has been related to addictive behaviors previously, an intronic (intron 8 VNTR) and a 40 bp VNTR located in the 3′UTR. A few studies found positive association between the 40 bp VNTR and cocaine dependence [86], ADHD [87] and alcoholism [88]. Here we did not find any positive relationship between either the 40 bp VNTR or the intron 8 VNTR of the SLC6A3 gene, with any of our calculation methods. The COMT exonic variant (Val158Met) has been associated with various psychiatric disorders (for a review see: Hosak, 2007 [89]), but no positive findings were obtained here with heroin abuse either with single marker or with multilevel analysis.

As all genetic association studies, the present one also has its limitations. Type I error of false positive results might arise from the relatively small sample size and from population heterogeneity. Our Hungarian sample seems to be homogeneous, where the genotype frequencies in the control group correspond to Hardy-Weinberg equilibrium (see: Table S1). Although family-based analyses are often preferred in order to avoid population stratification, but this approach is more difficult in case of subjects with substance abuse as many of these patients are not in touch with their families. Trying to recruit and searching for patients with complete and co-operating families might result in a drop out of the most serious cases, thus distorting the results [90]. A further limitation of our study is that the history of drug use was collected from self-reported data without any urine test for drugs. Additionally, we did not exclude cigarette smokers from our study since there is a high rate of smoking among heroin dependent patients despite the fact that the ANKK1 *Taq*IA A1 allele has been associated with smoking [91], [92]. Therefore, replications on other homogenous populations, as well as extended studies using a larger number of subjects would be necessary to further confirm the role of the −521 C/T, *Taq*IA and *Taq*IB polymorphisms in substance dependence.

Although numerous groups studied the individual effects of the dopaminergic candidate genes, little effort was done so far to reveal the interactions among these genetic variations. The main advantage of our study is the complex approach of the dopaminergic candidate genes applying multilevel analysis besides the single-marker methods to identify interactions and further contributing factors in the context of heroin dependence. As a result, the applied Bayesian method confirmed the relevance of DRD2 *Taq*IB SNP in heroin dependence and revealed a new interaction partner in the DRD4 promoter, the −615 A/G SNP modulating through the −521 C/T SNP. Further studies are in progress to apply these models for other, non-dopaminergic candidate genes of substance abuse.

## Supporting Information

Figure S1 **The summary of our findings.** The results of the BN-BMLA method are shown in the inner dark grey ring. The height of the red columns corresponding to each variable represents the probability that the variable is present in the Markov blanket of the target variable. The variables are grouped in the outer circle based on their respective genes. The interconnections in the center show the interaction and redundancy scores of the pairs of variables, where the edge thickness shows the effect’s strength, while red corresponds to interactions, and blue shows the redundancies.
(TIF)

Table S1 **The complete list of the VNTR genotypes including all DRD4 and DAT variants.**
(XLS)

Table S2 **Haplotype frequencies of the Taq SNPs in the DRD2 gene.**
(XLS)

Table S3 **Haplotype frequencies of the DRD4 promoter SNPs in controls and heroin dependent patients.**
(XLS)
