# Supplementary figures and images for: Multivariate Analysis of Dopaminergic Gene Variants as Risk Factors of Heroin Dependence
Source: PLoS One. 2013 Jun 28;8(6):e66592. doi: 10.1371/journal.pone.0066592 (PMC3696122; doi:10.1371/journal.pone.0066592)

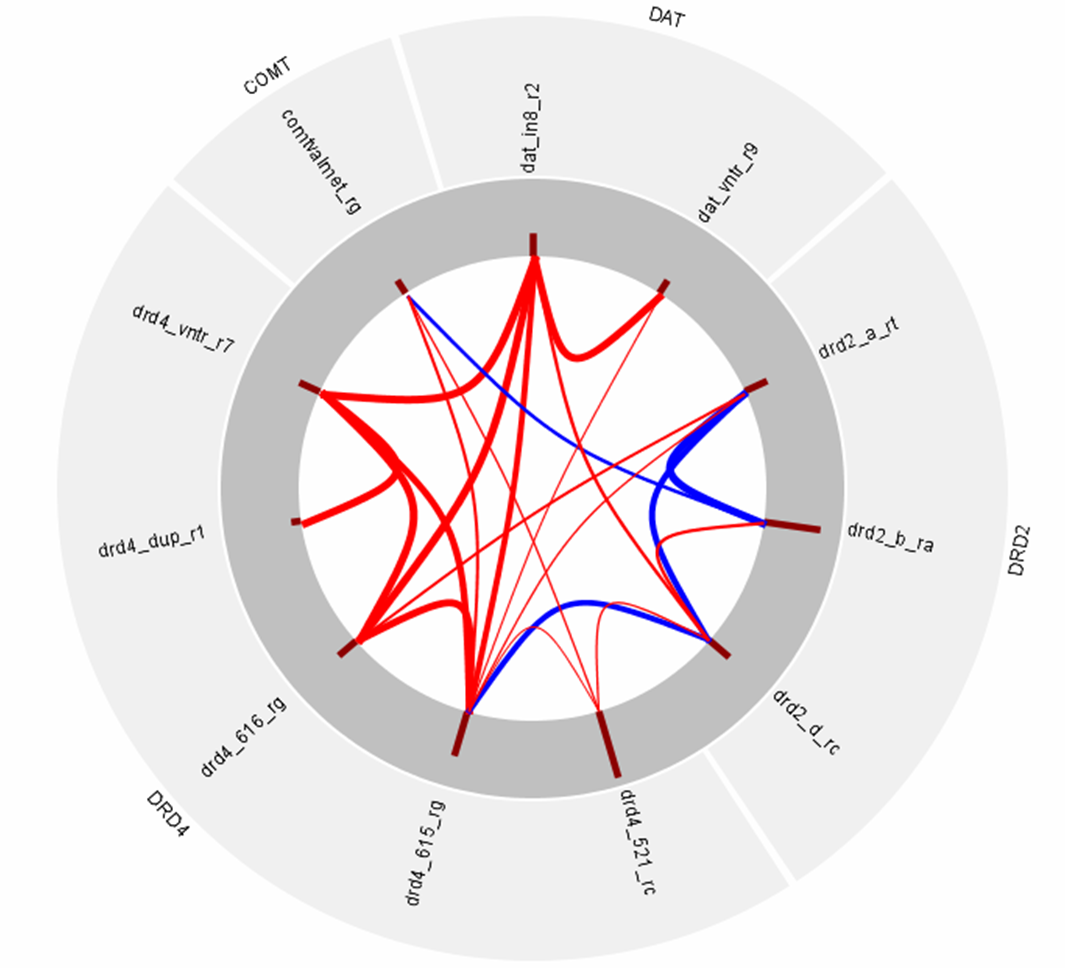

Supplement: Figure S1 — The summary of our findings. The results of the BN-BMLA method are shown in the inner dark grey ring. The height of the red columns corresponding to each variable represents the probability that the variable is present in the Markov blanket of the target variable. The variables are grouped in the outer circle based on their respective genes. The interconnections in the center show the interaction and redundancy scores of the pairs of variables, where the edge thickness shows the effect’s strength, while red corresponds to interactions, and blue shows the redundancies. (TIF) [file pone.0066592.s001.tif]
